# Antiviral Efficacy of Flavonoids against Enterovirus 71 Infection in Vitro and in Newborn Mice

**DOI:** 10.3390/v11070625

**Published:** 2019-07-07

**Authors:** Wenwen Dai, Jinpeng Bi, Fang Li, Shuai Wang, Xinyu Huang, Xiangyu Meng, Bo Sun, Deli Wang, Wei Kong, Chunlai Jiang, Weiheng Su

**Affiliations:** 1 National Engineering Laboratory for AIDS Vaccine, School of Life Sciences, Jilin University, Changchun 130012, China; 2 Key Laboratory for Molecular Enzymology and Engineering of the Ministry of Education, School of Life Sciences, Jilin University, Changchun 130012, China

**Keywords:** enterovirus 71, flavonoid, isorhamnetin, antiviral efficacy, inhibition of cytopathic effects, survival rate

## Abstract

Enterovirus 71 (EV71) infection is known to cause hand, foot, and mouth disease (HFMD), which is associated with neurological complications; however, there is currently no effective treatment for this infection. Flavonoids are a large group of naturally occurring compounds with multiple bioactivities, and the inhibitory effects of several flavonoids against EV71 have been studied in cell cultures; however, to date, there are no reported data on their effects in animal models. In this study, we confirmed the in vitro activities of eight flavonoids against EV71 infection, based on the inhibition of cytopathic effects. Moreover, these flavonoids were found to reduce viral genomic RNA replication and protein synthesis. We further demonstrated the protective efficacy of these flavonoids in newborn mice challenged with a lethal dose of EV71. Apigenin, luteolin, kaempferol, formononetin, and penduletin conferred survival protection of 88.89%, 91.67%, 88.89%, 75%, and 66.67%, respectively, from the lethal EV71 challenge. In addition, isorhamnetin provided the highest mice survival protection of 100% at a dose of 10 mg/kg. This study, to the best of our knowledge, is the first to evaluate the in vivo anti-EV7l activities of multiple flavonoids, and we accordingly identified flavonoids as potential leading compounds for anti-EV71 drug development.

## 1. Introduction

Hand, foot, and mouth disease (HFMD) primarily affects infants and children younger than 5 years of age, with symptoms such as fever, blisters, and rashes on the skin [1]. Although the disease is generally self-limiting, severe neurological manifestations induced by the causal agent enterovirus 71 (EV71) can occur [2]. EV71 is a neurotropic single-stranded positive-sense RNA virus, belonging to the Picornaviridae family. Infection with this virus may lead to acute central nervous system complications, including meningitis, encephalitis, poliomyelitis-like paralysis, neurogenic pulmonary edema, and even death, accounting for 80% of severe and 93% of fatal HFMD cases [3,4,5]. Since its first isolation in 1969, EV71 has been the source of numerous outbreaks worldwide, particularly in the Asia-Pacific region [6], and EV71 infection is accordingly considered a threat to public health.

Although two inactivated EV71 vaccines were approved in 2015 in mainland China for the prevention of severe HFMD, the lack of an established antiviral treatment for EV71 infection still remains a problem [7]. Supportive treatment to alleviate symptoms is commonly used in cases of severe EV71 infections [8]. Other than symptomatic treatment, intravenous immunoglobulin, interferon-alpha, and corticosteroids are used clinically [9,10]; however, the efficacy and safety of such treatments require further evaluation. Although a number of antiviral agents, including lactoferrin, pleconaril, shRNA, and rupintrivir, have shown demonstrable efficacy against EV71 infection, both in vitro and in vivo, none of them have been approved for clinical use [11,12,13]. Therefore, there is still an urgent need to identify pharmacological agents that are sufficiently effective in treating EV71 infection.

Flavonoids, characterized by a C6-C3-C6 carbon skeleton, constitute a large group of naturally occurring polyphenolic compounds that are ubiquitously distributed in fruits, vegetables, tea, soy foods, and herbs [14]. As flavonoids are dietary components with low toxicity, there have been numerous studies on their potential therapeutic benefits, with reported antioxidative, anti-inflammatory, anticancer, antibacterial, and antiviral properties [15,16]. The in vitro antiviral activities and putative mechanisms of several flavonoids against EV71 have been reported [17,18,19,20,21,22]. However, given that the results were obtained using different evaluation systems, it is difficult to compare the activities. Moreover, to the best of our knowledge, there is no record of studies that have examined the in vivo antiviral efficacy of flavonoids against EV71, which accordingly currently limits the further use of flavonoids as potential anti-EV71 agents. Therefore, there is an imperative to study the anti-EV71 activities of flavonoids, using a common evaluation system both in vitro and in vivo.

In this study, we examined twenty flavonoids, from which we initially screened out eight (apigenin, luteolin, kaempferol, quercetin, isorhamnetin, formononetin, chrysosplenetin, and penduletin) that were active against EV71 in cultured cells, and subsequently studied their antiviral efficacy against EV71, both in vitro and in vivo. Among these, isorhamnetin was identified for the first time as an inhibitor of EV71. All of the eight flavonoids were found to protect newborn BALB/c mice from a lethal EV71 challenge. On the basis of our observations, we conclude that flavonoids are promising antiviral agents against EV71 with considerable efficacy.

## 2. Materials and Methods

### 2.1. Compounds, Cells, Viruses, and Animals

Flavonoid standards (apigenin, luteolin, diosmetin, tangeretin, nobiletin, galangin, kaempferol, quercetin, myricetin, isorhamnetin, silibinin, liquiritigenin, bavachinin, taxifolin, dihydromyricetin, daidzein, formononetin, epicatechin, chrysosplenetin, and penduletin) with purities higher than 98% determined by HPLC, were purchased from Meilun Biotech Co., Ltd (Dalian, China). The compounds were dissolved in dimethyl sulfoxide (DMSO) for storage.

Human embryonic kidney cells stably overexpressing the human scavenger receptor class B, member 2 (SCARB2) (293S cells) were cultured in Dulbecco’s modified Eagle’s medium (DMEM; Invitrogen, Carlsbad, CA, USA) supplemented with 10% fetal bovine serum (FBS; Invitrogen) (DMEM-10% FBS) with the addition of 1.25 μg/mL puromycin (Sigma, St. Louis, MO, USA) at 37 °C in a 5% CO2 atmosphere, as previously reported [23].

The wild-type EV71 C4 strain (WT-EV71) (Gene Bank accession no. KJ508817) was obtained from the Chinese Center for Disease Control and Prevention. Its propagation and titration were performed in 293S cells. The EV71 pseudovirus harboring a luciferase reporter gene (EV71-luc) was previously constructed by our group [24].

Female breeder BALB/c mice were obtained from the Changchun Institute of Biological Products. Newborn BALB/c mice within 24 h of birth were used for in vivo experiments. Protocols for mouse experiments were approved by the Laboratory Animal Ethics Committee of School of Life Science, Jilin University (Approval number 2015-nsfc017).

### 2.2. Cytopathic Effect (CPE) Inhibition Assay

The antiviral activities of the flavonoids against EV71 were determined using CPE inhibition assays. Briefly, 90% confluent 293S cells were infected with WT-EV71 (MOI = 0.04, which was determined to cause 100% CPE and 60% viability loss in 293S cells) in the presence of serially diluted flavonoid compounds in DMEM-2% FBS. At 48 h post-infection, cell viabilities were determined using the Cell Titer-Glo® reagent (Promega, Madison, WI, USA) and a PerkinElmer VICTORTM X2 microplate reader (Waltham, MA, USA) as reported [25]. The inhibition percentage of CPE was calculated as follows:

(T − V)/(C − V) × 100%,

where T, V, and C are the luminescence intensities of drug-treated cells, virus control, and cell control, respectively. The 50% effective concentrations (EC50) were calculated by regression analysis of the dose-response curves. This experiment was repeated twice with *n* = 3 replicates in each repeat.

### 2.3. Cytotoxicity Assay

The cytotoxic effects of the flavonoids on 293S cells were determined using a Cell Titer-Glo® Luminescent cell viability assay. Briefly, 293S cells in 96-well plates were grown to 90% confluency, to which we added DMEM supplemented with 2% FBS (DMEM-2% FBS) containing serially diluted compounds. After incubation for 48 h, cell viabilities were assayed as described in Section 2.2. Cell viability percentages were calculated as ratios of the luminescence intensities of drug-treated cells to that of the untreated cell control. The 50% cytotoxic concentrations (CC50) were calculated by regression analysis of the dose-response curves. Selective index values were calculated as ratios of CC50 to EC50. This experiment was repeated twice with *n* = 3 replicates in each repeat.

### 2.4. Quantitative Reverse Transcription-Polymerase Chain Reaction (qRT-PCR)

We infected 90% confluent 293S cells with WT-EV71 (MOI = 1) in the presence of the flavonoid compounds at their optimal concentrations, as determined in the CPE inhibition assay (50 μM apigenin, 25 μM luteolin, 200 μM kaempferol, 3.13 μM quercetin, 100 μM isorhamnetin, 25 μM formononetin, 2.5 μM chrysosplenetin, and 0.63 μM penduletin). After one replicative lifecycle at 16 h post-infection [26], viral RNA was extracted from the cell cultures after freeze–thaw cycles using a TIANamp RNA Kit for Virus Detection (TIANGEN Biotech Co., Ltd, Beijing, China). qRT-PCR was conducted with the One Step SYBR^®^ PrimeScript^™^ RTPCR Kit II (Takara Bio, Otsu, Japan) using the Bio-Rad CFX96 system (Hercules, CA, USA). EV71 RNA was detected using sense primer 5′-TCCTCCGGCCCCTGA-3′ and antisense primer 5′-AATTGTCACCATAAGCAGCCA-3′, targeting the 5′ UTR as previously reported [27]. This experiment was repeated twice with *n* = 3 replicates in each repeat.

### 2.5. EV71-luc Based Viral Protein Synthesis Assay

We infected 90% confluent 293S cells with 200 CCID50 (50% cell culture infective dose) of EV71-luc in the presence of the flavonoid compounds at their optimal concentrations as determined in the CPE inhibition assay as previously reported [18]. After one replicative lifecycle at 16 h post-infection [26], luminescence intensities were assayed using Bright-Glo^™^ reagent (Promega) and a PerkinElmer VICTORTM X2 microplate reader (Waltham). This experiment was repeated twice with *n* = 3 replicates in each repeat.

### 2.6. Protective Efficacy against EV71 in Newborn Mice

Newborn BALB/c mice were infected with 600,000 TCID50 (lethal dose) of WT-EV71 by intracranial inoculation, within 24 h of birth [28]. Infected mice were intraperitoneally injected with different doses of flavonoids in sterile PBS supplemented with 10% DMSO or with sterile PBS supplemented with 10% DMSO (infected control) daily for seven consecutive days, starting from 2 h post-infection. Uninfected mice injected with sterile PBS supplemented with 10% DMSO were treated as uninfected controls. The mice were monitored daily for signs of morbidity and mortality for 16 days. Severity of illness was evaluated using a graded score (0, healthy; 1, slow movement; 2, weakness in hind limbs; 3, paralysis in a single limb; 4, paralysis in two limbs; and 5, death) as previously reported [29]. In addition, body weight was determined and normalized as

Wn/Wo,

where Wn and Wo are the body weights of the newborn mice on day n and day 0, respectively. These experiments were repeated twice with *n* = 15 newborn mice in each repeat.

### 2.7. Statistical Analysis

Experiments were performed in triplicate with the values expressed as the mean ± SD. A log-rank test (Mantel–Cox) and a one-way ANOVA test were performed respectively to compare the differences in the survival rates and other values between test groups and virus control groups. Statistical significance is denoted by asterisks and indicated correspondingly in the figures: * *p* < 0.05, ** *p* < 0.01, *** *p* < 0.001.

## 3. Results

### 3.1. Flavonoids Protected Cells from EV71-induced CPE

**Figure 1:**
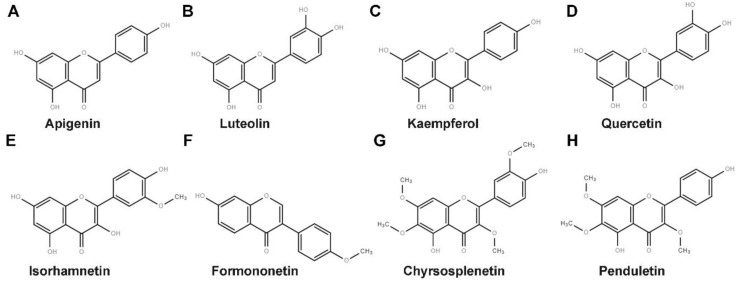
Chemical structures of (**A**) Apigenin (**B**) Luteolin (**C**) Kaempferol (**D**) Quercetin (**E**) Isorhamnetin (**F**) Formononetin (**G**) Chrysosplenetin and (**H**) Penduletin.

**Figure 2:**
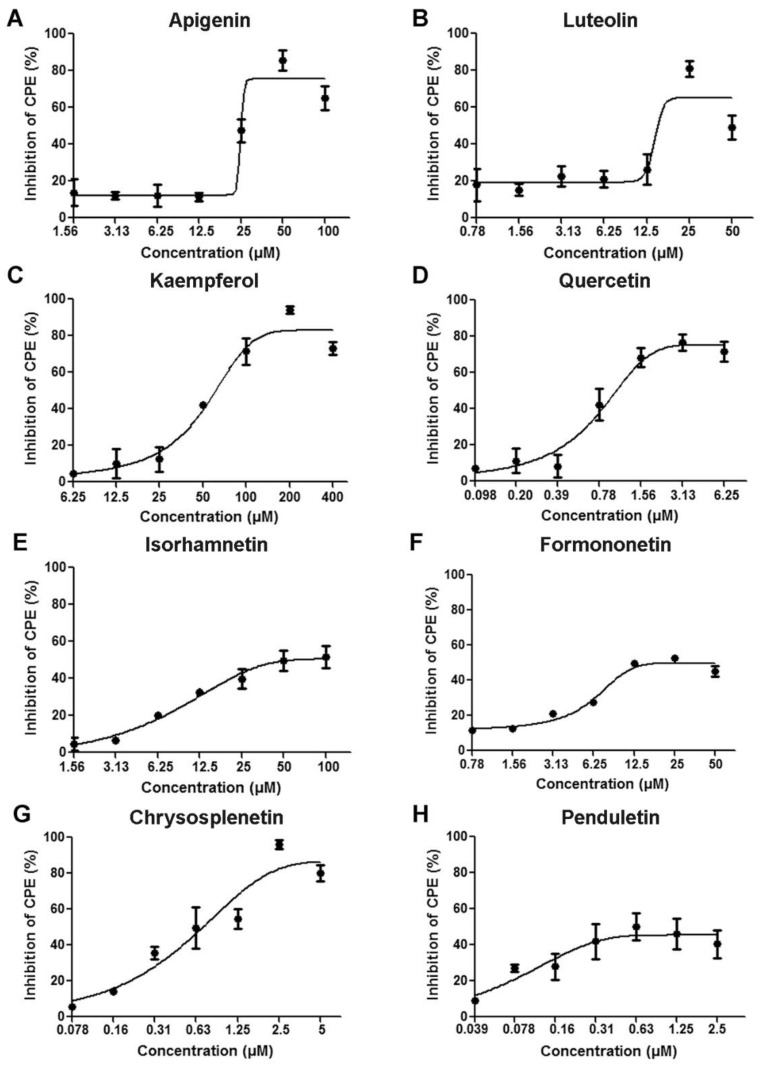
Effects of (**A**) Apigenin (**B**) Luteolin (**C**) Kaempferol (**D**) Quercetin (**E**) Isorhamnetin (**F**) Formononetin (**G**) Chrysosplenetin and (**H**) Penduletin on EV71-induced CPE. 293S cells were infected with WT-EV71 (MOI = 0.04) and simultaneously treated with serially diluted flavonoids. After incubation for 48 h, cell viabilities were measured to calculate the inhibitory percentages of EV71-induced CPE. *N* = 3 replicates in each concentration.

**Figure 3:**
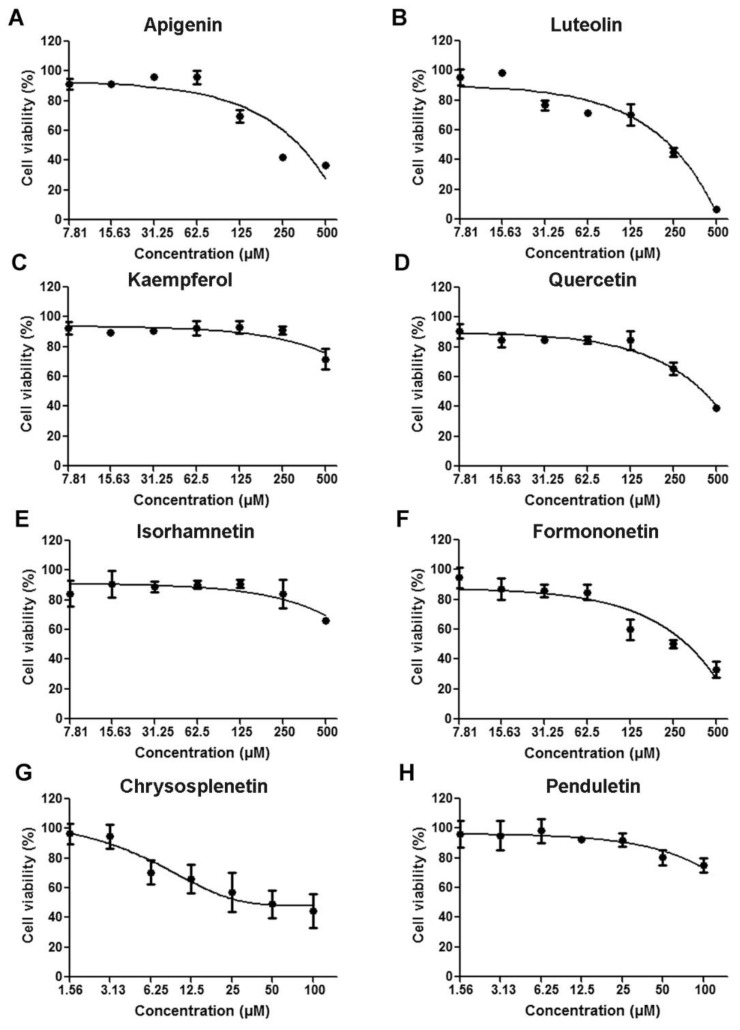
Cytotoxicity of (**A**) Apigenin (**B**) Luteolin (**C**) Kaempferol (**D**) Quercetin (**E**) Isorhamnetin (**F**) Formononetin (**G**) Chrysosplenetin and (**H**) Penduletin on 293S cells. 293S cells were treated with serially diluted flavonoids for 48 h, after which the cell viabilities were measured and compared to that of the cell control. *N* = 3 replicates in each concentration.

We initially screened twenty flavonoids to identify those with antiviral activities against EV71 in 293S cells based on CPE inhibition assays (data not shown). We accordingly identified eight flavonoids (Figure 1) with inhibitory rates higher than 50% as effective candidates and these were selected for further evaluation. As there is currently no specific drug against EV71 infection for clinical use, no proper one was available to be used as a positive control [9]. We set up only a cell control and a virus infected control in the in vitro assays. As shown in Figure 2, the selected flavonoids inhibited EV71-induced CPE in a dose-dependent manner. The highest inhibitory rates were as follows: 85.65% ± 5.57% for apigenin at 50 μM, 80.84% ± 4.39% for luteolin at 25 μM, 94.13% ± 2.17% for kaempferol at 200 μM, 76.56% ± 4.71% for quercetin at 3.13 μM, 51.58% ± 5.78% for isorhamnetin at 100 μM, 52.67% ± 0.93% for formononetin at 25 μM, 96.20% ± 2.45% for chrysosplenetin at 2.5 μM, and 50.21% ± 7.49% for penduletin at 0.63 μM. As shown in Figure 3, no significant reduction in cell viability was detected at the concentrations used in the antiviral activity assays, indicating that the inhibitory effects of the flavonoids were not due to cytotoxicity. As shown in Table 1, 50% effective concentration (EC50) and 50% cytotoxic concentration (CC50) values were calculated by regression analysis of the dose-dependent curves. In addition, selective index (SI) values representing the ratio of CC50/EC50 were also calculated. All flavonoids showed SI values higher than 4, which indicated that they are suitable for drug development [30]. Collectively, these results indicate that all eight flavonoids are safe and effective antivirals that protect cells from EV71 infection.

### 3.2. Flavonoids Caused Reduced EV71 Replication in Cells

**Figure 4:**
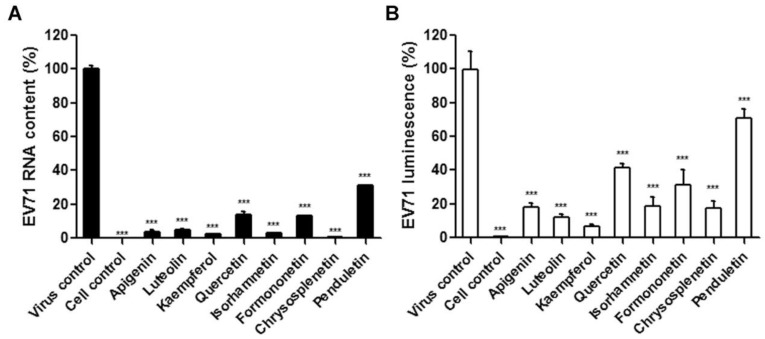
Effects of the flavonoids on EV71 RNA replication (**A**) and protein (**B**) synthesis. (**A**) 293S cells were infected with WT-EV71 (MOI = 1) in conjunction with the treatment of the flavonoids at optimal concentrations. At 16 h post-infection, EV71 RNA was extracted from the cell cultures and subjected to qRT-PCR. (**B**) 293S cells were infected with EV71-luc (200 CCID50) in conjunction with the treatment of the flavonoids at optimal concentrations. At 16 h post-infection, EV71 protein synthesis was monitored via measurement of the luminescence intensity. *N* = 3 replicates for each compound. A one-way ANOVA test was performed to compare the differences between test groups and the virus control with the statistical significance denoted by asterisks, *** *p* < 0.001.

To further confirm the inhibitory effects of the selected flavonoids against EV71, viral RNA content and protein synthesis in a single lifecycle at 16 h post-infection were determined by qRT-PCR and luminescence of EV71-luc, respectively. As shown in Figure 4, significant reductions in both EV71 RNA and protein synthesis were observed in flavonoids-treated cells, indicating that all eight flavonoids are effective antivirals that reduced EV71 replication in cells.

### 3.3. Flavonoids Protected Newborn BALB/c Mice from EV71-induced Lethality

Having confirmed the potential of flavonoids against EV71 in vitro, we next evaluated their protective efficacy in newborn mice as previously reported [28]. Specifically, newborn BALB/c mice were intracranially infected with a lethal dose of EV71, within 24 h of birth, followed by seven consecutive days of intraperitoneal inoculation of flavonoids at different doses, which were determined by their EC50 values in vitro. As the antiviral effect in vivo is correlated with that of in vitro, EC50 values in cell cultures could provide a reference for the dosage choice in mice. In our previously published study, Retro-2^cycl^ with the EC50 value of 12.56 μM showed good protective efficacy in mice against EV71 infection at the dosages of 2 mg/kg and 10 mg/kg [28]. Accordingly, apigenin, kaempferol, and isorhamnetin with higher EC50 values were administered at doses of 10 mg/kg and 50 mg/kg, whereas luteolin, quercetin, and formononetin with intermediate EC50 values were administered at doses of 2 mg/kg and 10 mg/kg, and chrysosplenetin and penduletin with lower EC50 values were administrated doses of 1 mg/kg and 5 mg/kg. As there is currently no specific drug against EV71 infection for clinical use, no proper one was available to be used as a positive control [9]. We set up only groups of uninfected mice and infected mice without drug treatment as controls in the in vivo assays. As neonatal mice were used in the present study, it was difficult to collect the blood samples without death because they are too small to manipulate. Besides, the serum amount collected would be too small to measure. Therefore, the parameters of serum were not detected in the present study.

**Figure 5:**
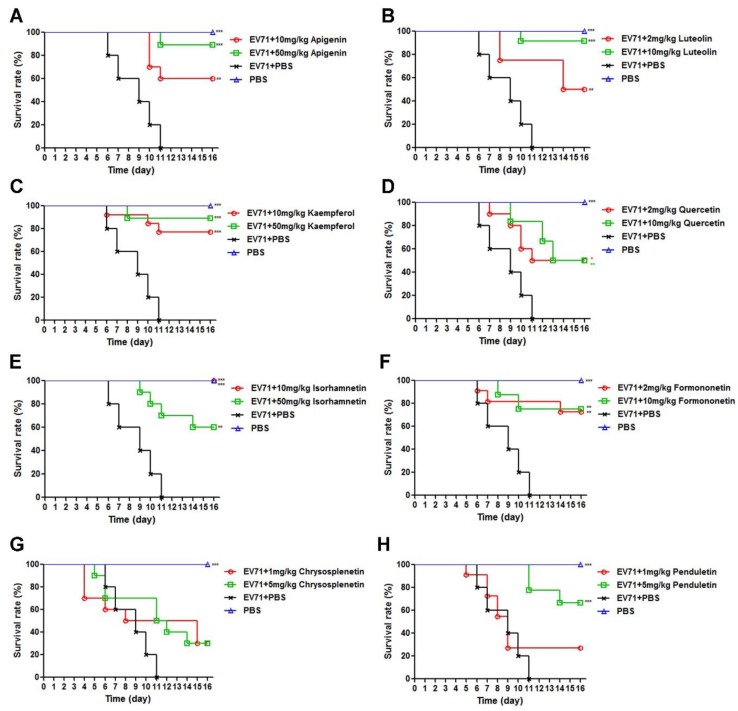
Protective efficacy of (**A**) Apigenin (**B**) Luteolin (**C**) Kaempferol (**D**) Quercetin (**E**) Isorhamnetin (**F**) Formononetin (**G**) Chrysosplenetin and (**H**) Penduletin on the survival rates of newborn mice challenged with a lethal dose of EV71. Newborn BALB/c mice were intracranially inoculated with 600,000 TCID50 WT-EV71 within 24 h of birth, followed by intraperitoneal injections of flavonoids in 10% DMSO-PBS at different doses for seven consecutive days. Survival rates were monitored for 16 days post-infection. *N* = 15 newborn mice in each group. A log-rank test (Mantel–Cox) test was performed to compare the differences between test groups and the virus control with the statistical significance denoted by asterisks, * *p* < 0.05, ** *p* < 0.01, *** *p* < 0.001.

**Figure 6:**
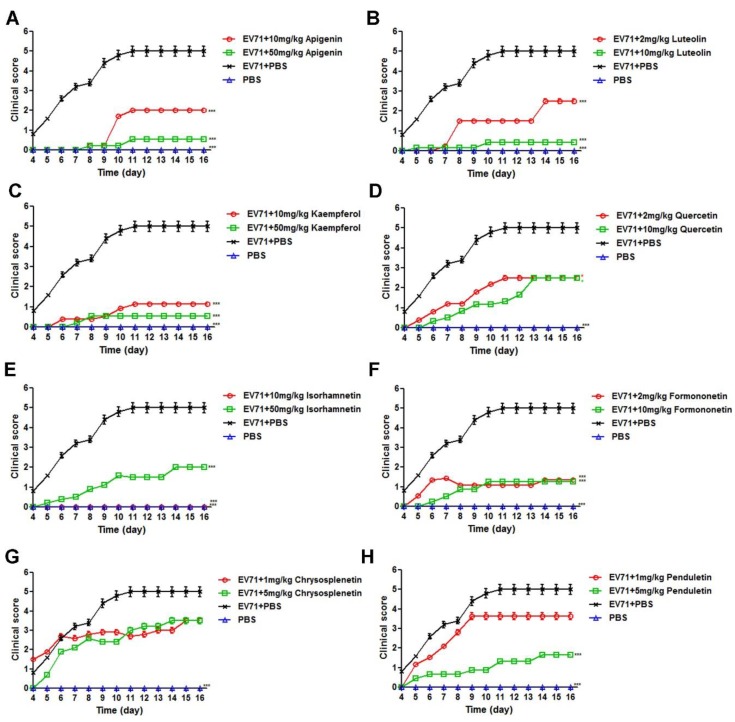
Effects of (**A**) Apigenin (**B**) Luteolin (**C**) Kaempferol (**D**) Quercetin (**E**) Isorhamnetin (**F**) Formononetin (**G**) Chrysosplenetin and (**H**) Penduletin on the clinical scores of newborn mice challenged with a lethal dose of EV71. Newborn BALB/c mice were intracranially inoculated with 600,000 TCID50 WT-EV71 within 24 h of birth, followed by intraperitoneal injections of flavonoids in 10% DMSO-PBS at different doses for seven consecutive days. Clinical scores (0, healthy; 1, slow movement; 2, weakness in hind limbs; 3, paralysis in a single limb; 4, paralysis in two limbs; and 5, death) were monitored for 16 days post-infection. N = 15 newborn mice in each group. A one-way ANOVA test was performed to compare the differences between test groups and the virus control with the statistical significance denoted by asterisks, * *p* < 0.05, *** *p* < 0.001.

**Figure 7:**
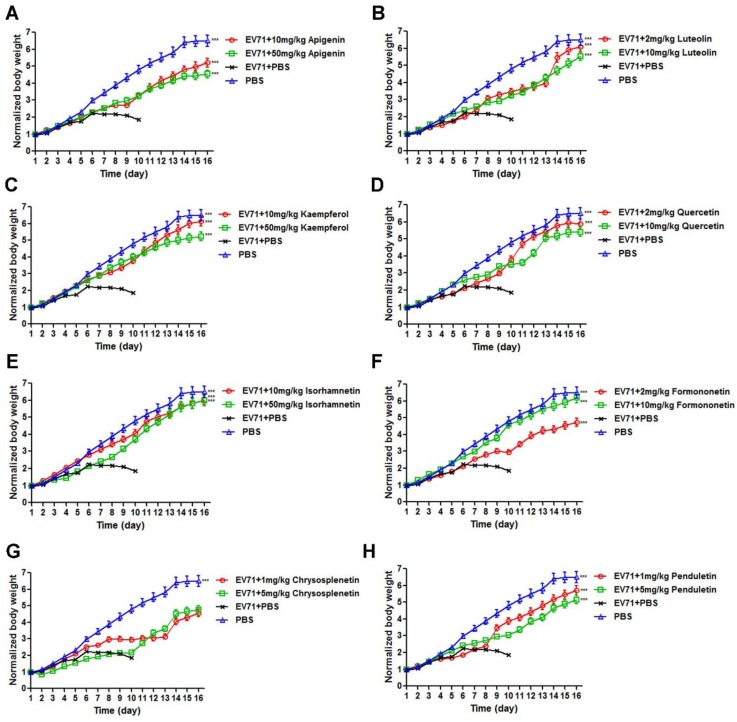
Effects of (**A**) Apigenin (**B**) Luteolin (**C**) Kaempferol (**D**) Quercetin (**E**) Isorhamnetin (**F**) Formononetin (**G**) Chrysosplenetin and (**H**) Penduletin on the body weights of newborn mice challenged with a lethal dose of EV71. Newborn BALB/c mice were intracranially inoculated with 600,000 TCID50 WT-EV71 within 24 h of birth, followed by intraperitoneal injections of flavonoids in 10% DMSO-PBS at different doses for 7 consecutive days. Body weights were monitored for 16 days post-infection. *N* = 15 newborn mice in each group. A one-way ANOVA test was performed to compare the differences between test groups and the virus control with the statistical significance denoted by asterisks, *** *p* < 0.001.

As shown in Figure 5, all eight selected flavonoids protected the infected mice from the EV71 challenge as compared to infected control mice, which all died within 11 days without drug intervention. Apigenin, luteolin, kaempferol, quercetin, formononetin, and penduletin exhibited better protective efficacy at higher dosages. However, the survival rates of isorhamnetin and chrysosplenetin did not follow the dose-dependent manner. For the dose–response relationship, the drug efficacy had positive correlation with the dosage in a certain range, where higher dosages of isorhamnetin and chrysosplenetin outside the range might be less protective against EV71, and this tendency was also reported in other publications [29]. Besides, the lower protective rate of higher dosages of isorhamnetin and chrysosplenetin might also be explained by the low solubility of the compounds in PBS, which might have cause aggregation and reduced bioavailability. The best survival rates were as follows: 88.89% for apigenin at 50 mg/kg, 91.67% for luteolin at 10 mg/kg, 88.89% for kaempferol at 50 mg/kg, 50% for quercetin at 10 mg/kg, 100% for isorhamnetin at 10 mg/kg, 75% for formononetin at 10 mg/kg, 30% for chrysosplenetin at 5 mg/kg and 1 mg/kg, and 66.67% for penduletin at 5 mg/kg. Among these flavonoids, isorhamnetin showed the best protective efficacy of 100% at the optimal dose of 10 mg/kg. As shown in Figure 6, clinical scores showed the same trend as survival rates. All flavonoids reduced the clinical scores of infected mice to a certain extent, whereas the values of infected control mice without drug intervention reached 5 on day 11. Moreover, infected mice treated with 10 mg/kg isorhamnetin showed no adverse symptoms during the entire period of observation. We also recorded body weights in order to examine the inhibitory efficacy of the flavonoids on EV71-induced weight loss. As shown in Figure 7, body weights of the drug-treated mice all increased with time, whereas those of infected control mice showed a decrease from day 6 until the end of the period of observation. As the body weight values were calculated only in the surviving mice, all these flavonoids at both dosages exhibited nearly the same trend. In addition, the body weight of infected mice treated with 10 mg/kg isorhamnetin showed no significant difference from those of uninfected mice, thereby indicating that isorhamnetin is non-toxic. Therefore, we demonstrated that the selected flavonoids also effectively protected newborn mice from the EV71 challenge and that isorhamnetin at the dose of 10 mg/kg showed the best protective rate of 100%.

## 4. Discussion

EV71 is the etiologic pathogen for HFMD, particularly those associated with neurological syndromes [1]. It has spread worldwide and led to public health problems [2]; however, to date there are no effective therapeutic measures available. Plant-derivative flavonoids are a large group of naturally occurring compounds with broad biological activities [31], several of which the antiviral effects and underlying mechanisms against EV71 in cell cultures have been reported. Among these flavonoids, apigenin has been reported to inhibit EV71 replication by disrupting viral RNA association with trans-acting factors and modulating the cellular JNK pathway [17]. In our previous study, we demonstrated that luteolin targets the post-attachment stage of EV71 [18]. Kaempferol has been reported to inhibit EV71 replication and IRES activity via FUBP and HNRP proteins [19]. Formononetin has been reported to inhibit EV71 replication by regulating COX/PGE2 expression [21]. Chrysosplenetin and penduletin have been demonstrated to act on the early post-attachment stage of the EV71 lifecycle [22]. Quercetin has also been found to show inhibitory effects on EV71 [20]. However, all these results pertain to the inhibitory effects in cell cultures. Given that the demonstration of antiviral efficacy in animal models is an important basis for drug development, we evaluated the protective efficacy of flavonoids against EV71 in newborn mice.

In the present study, we initially screened a selection of twenty flavonoids and identified eight with EV71 induced-CPE inhibitory percentages higher than 50%, among which the antiviral activity of isorhamnetin, a known poliovirus inhibitor [32], against EV71 was identified for the first time. We also confirmed the inhibitory effects of the other seven flavonoids against EV71. The EC50 values were determined using the same evaluation system, thereby providing a reference for selecting appropriate doses of the flavonoids for use in in vivo experiments. Moreover, the SI values obtained for these flavonoids were all higher than 4, indicating that these flavonoids are effective inhibitors of EV71 in cell cultures without any obvious cytotoxicity [30]. On the basis of the in vitro results, we further examined the protective efficacy of flavonoids against a lethal EV71 challenge in newborn BALB/c mice as previously reported [28]. Each of the assessed flavonoids was demonstrated to be an effective inhibitor of EV71 in newborn mice, as manifested by the improved survival rates, decreased clinical scores, and altered weight loss. In particular, isorhamnetin at the dose of 10 mg/kg showed a 100% protective rate, and is accordingly considered to be the most promising candidate for development as an antiviral against EV71.

To date, two antiviral agents against EV71, namely, pleconaril and rupintrivir, have undergone clinical trials [33]. In published studies, rupintrivir has been demonstrated to enhance the survival rate of EV71-infected 2-day-old suckling mice from 38.5% to 90.9% when administrated at 0.1 mg/kg once daily for 10 days [13]. Similarly, pleconaril has been demonstrated to enhance the survival rate of EV71-infected 1-day-old suckling mice from 20% to 80% when administrated at 80 mg/kg once daily for 5 days [12]. In the present study, we found that seven of eight assessed flavonoids have significant protective efficacy against a lethal EV71 challenge in newborn mice with survival rates of 50% or higher, with 10 mg/kg isorhamnetin conferring 100% protection, 10 mg/kg luteolin conferring 91.67% protection, 50 mg/kg apigenin and kaempferol conferring 88.89% protection, 10 mg/kg formononetin conferring 75% protection, 5 mg/kg penduletin conferring 66.67% protection, and 10 mg/kg quercetin conferring 50% protection. Accordingly, studies on antivirals against EV71 in animal models, including the present study, might potentially heighten the prospect of anti-EV71 agent development. Furthermore, as approximately 9,000 flavonoids have been described to date, identification of the antiviral efficacy of flavonoids against EV71 both in vitro and in vivo would provide numerous potential leading compounds for anti-EV71 drug development [34]. Moreover, flavonoids with enhanced antiviral effects could also be developed through further structural modification.

Some of the flavonoids included in the present study have also been found to show antiviral potential against picornaviruses other than EV71. Coxsackievirus A16 (CVA16) infection can be inhibited by luteolin, quercetin, and kaempferol [18,20], whereas coxsackievirus B2 (CVB2), CVB3, and CVB6 infections can be inhibited by formononetin [21]. Similarly, poliovirus (PV) infection can be inhibited by luteolin, quercetin, and isorhamnetin [32,35], rhinovirus infection can be inhibited by quercetin [36], and foot and mouth disease virus (FMDV) infection can be inhibited by apigenin [37]. Moreover, these flavonoids have also been reported as inhibitors of other single-stranded positive-sense RNA viruses. Apigenin, luteolin, kaempferol, and quercetin show inhibitory effects against the chikungunya virus (CHIK-V) [38,39,40]; apigenin, quercetin, and isorhamnetin show inhibitory effects against the hepatitis C virus (HCV) [41,42,43]; luteolin and quercetin show inhibitory effects against the dengue virus [39,44]; luteolin and kaempferol show inhibitory effects against the Japanese encephalitis virus (JEV) [45,46]; and isorhamnetin shows inhibitory effect against the Zika virus [47].

Although flavonoids show considerable antiviral activities, the detailed underlying mechanisms and safety issues remain to be explored. Moreover, structural modifications based on leading compounds with optimal activity, such as isorhamnetin, are still needed to obtain better candidates for antiviral development.

Taken together, in the present study, we confirmed the anti-EV71 activities of eight flavonoids in vitro and demonstrated their in vivo protective efficacy in newborn mice for the first time. Furthermore, isorhamnetin at a dose of 10 mg/kg could confer the protection in 100% of mice from a lethal EV71 challenge. Our findings will therefore provide potentially useful data for the development of antiviral therapy for EV71 and other related viruses.

## Figures and Tables

**Table 1:** Cytotoxicity and antiviral activities of eight flavonoids against EV71 in 293S cells.

	Cytotoxicity	Antiviral Activity	
Compounds	CC_50_^a^ (μM)	EC_50_^b^ (μM)	SI ^c^
Apigenin	256.1	24.74	10.35
Luteolin	168.2	13.5	12.46
Kaempferol	>500	52.75	>9.48
Quercetin	444.2	1.2	370.17
Isorhamnetin	>500	60.7	8.24
Formononetin	238.9	12.5	19.11
Chrysosplenetin	48.97	0.68	72.01
Penduletin	>100	0.63	158.7

^a^ The concentration at which a compound causes 50% cytotoxicity. ^b^ The concentration at which a compound causes 50% inhibition of viral cytopathic effect. ^c^ The SI value represents the ratio of CC_50_/EC_50_ for each compound. Results are presented as mean values obtained from two independent experiments.
